# Contrast bias, not confirmation bias, shapes daily impression updating across social and non‐social targets

**DOI:** 10.1111/bjso.70123

**Published:** 2026-08-07

**Authors:** Yingjie Li, Jean Daunizeau, Sabine Geurts, Johan Karremans, Fred Hasselman

**Affiliations:** ^1^ Behavioural Science Institute Radboud University Nijmegen The Netherlands; ^2^ Research Office University of Cambridge Cambridge UK; ^3^ Paris Brain Institute (ICM) Paris France

**Keywords:** computational modelling, contrast bias, contrast effect, impression updating, social non‐social comparison

## Abstract

Previous literature on impression updating has paid extensive attention to confirmation bias *‐ the tendency to seek out, favour, interpret or remember information in a way that reinforces existing beliefs*. Fewer studies examined the *contrast effect/bias* in the sequential updating of impressions. Contrast bias *occurs when the difference between current beliefs and new information is exaggerated, leading to a stronger shift in evaluation than the new information alone would justify*. In this work, we conducted two Ecological Momentary Experiments to capture real‐world impression formation and updating on both social (e.g. an employee) and non‐social (e.g. a vacation house) targets. We also developed novel computational models of biased impression updating based on parametric distortions of Bayesian ideal observers. Across the two studies, we found that daily impression updating exhibited significant contrast bias, with negative contrast bias exerting a stronger influence than positive ones. Additionally, we found no evidence for differences in impression updating between social and non‐social targets. By focusing on within‐person impression updating over time, these findings reveal contrast bias as a core dynamic of impression updating, systematically shaping how impressions change in response to inconsistent information.

## INTRODUCTION

Forming and updating impressions of the social and non‐social environment is crucial for navigating daily life, making decisions and communicating effectively. An impression of a co‐worker as friendly and competent shapes how we engage with them; an impression of a city as disorganized affects how long we stay and what services we trust.

Research on impressions (e.g. of a person, object or event) distinguishes two related but distinct processes (Amodio, 2025). The first is impression *formation*: Initial encounters give rise to momentary evaluations and beliefs shaped by immediate experiences and preconceptions. For example, we might have a first impression of a co‐worker as confident and friendly based on their appearance. The second is impression *updating*: As more interactions occur, more information will be gathered (Cuddy et al., 2011; Fiske & Neuberg, 1990), for example, about the communication style and competencies of the co‐worker. All this new information will be used to either reinforce or alter the first impression.

The present work focuses on impression updating: the process by which existing impressions are revised in response to new information. Rather than reflecting a straightforward accumulation of evidence, impression updating depends on how new information is interpreted, represented and integrated with prior beliefs (Moskowitz et al., 2022; Van Overwalle & Labiouse, 2004). As a result, impression updating is a dynamic, biased and socially motivated process.

Impression updating may be shaped by positive and negative biases, which reflect the tendency to weight positive or negative information more strongly (Rozin & Royzman, 2001). The direction of this asymmetry depends on the trait domain. According to the schematic model of dispositional attribution, morality‐related traits show a negativity bias because a single immoral act strongly implies an immoral disposition, whereas many moral acts are needed to establish morality (Reeder & Brewer, 1979). Competence‐related traits show the opposite pattern, as competent individuals can still occasionally fail (Cuddy et al., 2011; Williams & Tiedens, 2016). Empirical work has confirmed these content‐dependent asymmetries: A negativity effect emerges for morality‐related information but a positivity effect for competence‐related information, with both effects moderated by evaluative extremity (Skowronski & Carlston, 1989; Wojciszke et al., 1993). Although these asymmetries were established primarily in the impression formation literature, they provide an important theoretical basis for understanding impression updating. If certain behaviours are more diagnostic of underlying traits, they should not only shape initial impressions but also carry greater weight when existing impressions are revised. Consistent with this idea, these asymmetries also affect the amount of evidence needed to form or reverse impressions: Positive traits are hard to confirm and easy to disconfirm, whereas negative traits are readily confirmed but require substantial counterevidence to reverse (Rothbart & Park, 1986).

Beyond biases tied to information valence, some biases are shaped by whether new information aligns with the current impression. Contextual cues can shift impressions: When these cues are integrated into the impression of the target, judgements assimilate towards them, but when they are treated as standards of comparison, judgements contrast away from them (Bless & Schwarz, 2010; Schwarz & Bless, 1992). For instance, studies have shown that initial information can anchor subsequent evaluations, leading to a stronger reliance on confirmatory evidence and discounting information that contradicts the initial belief (Furnham & Boo, 2011; Nickerson, 1998; Tversky & Kahneman, 1974). In such cases, updating may proceed by selectively discounting inconsistent information, thereby reinforcing the initial impression. Alternatively, contradictory information may be treated as particularly informative, triggering disproportionate updating and causing impressions to shift away from their initial direction (Mende‐Siedlecki & Todorov, 2016). These competing possibilities—confirmation versus contrast—make qualitatively different predictions about the temporal dynamics of impression updating, especially under repeated exposure to mixed information.

### CONFIRMATION VS. CONTRAST

Confirmation bias refers to the tendency to favour, search for, recall or interpret evidence in a way that aligns with one's existing beliefs or impressions (Hahn & Harris, 2014; Nickerson, 1998). Although confirmation effects may also arise from positive testing, whereby people ask about what they expect to be present rather than about its opposite, combined with a tendency for respondents to answer ‘yes’ (Klayman, 1995; McKenzie, 2004), the present work focuses on how information is integrated once it has been received. Confirmation effects have been explained in terms of motivational factors, including self‐enhancement, affiliation goals and argumentation needs. For instance, when people form impressions of their in‐group members, they tend to show confirmation bias because of affiliation goals—the goals to get along with the impression target (Sinclair et al., 2005; Ybarra et al., 2001). Similarly, when people evaluate themselves, it may come from the motivation to preserve a positive self‐image (Trouche et al., 2016). It has been argued that confirmation bias exists when people are preparing arguments to convince others or defend themselves (Mercier & Sperber, 2011), and its presence is not affected by the valence of the beliefs or impressions: when the initial impression is negative, people interpret new information in a negative way (Downey & Christensen, 2006; Gershman & Cikara, 2023; Richey et al., 1967). Similarly, when the initial impression is positive, people integrate new information in a way that reinforces this positivity (Sinclair et al., 2005; Ybarra et al., 2001). Confirmation bias has been found in various areas (Peters, 2022), including impression formation (Nickerson, 1998; Rabin & Schrag, 1999).

However, can the opposite of confirmation bias occur? Can contradictory information be overemphasized in impression updating? Research suggests that this can happen when the new information is surprising, salient and/or emotionally impactful (Antony et al., 2023; Kensinger, 2009; Van Overwalle & Labiouse, 2004). In this context, the new information would be emphasized when forming a general impression. This phenomenon can be seen as a variant of the contrast bias. Contrast bias occurs because the new information is treated as a separate standard of comparison, highlighting discrepancies with prior impressions (Schwarz & Bless, 1992). Contrast bias has been most extensively studied in contexts where different targets are compared, such as in performance evaluation (e.g. examiners give higher scores after seeing a weaker performance and lower scores after observing a higher performance; Yeates et al., 2015). Within a single target, the comparable phenomenon (new information being weighted against a prior reference point) has been examined under different theoretical labels, such as diagnosticity. For instance, some studies suggest that inconsistent information (namely, when new information does not align with the previous impression) triggers a stronger shift in impression, especially when the inconsistency is meaningful (Mende‐Siedlecki & Todorov, 2016). This is because such information is often seen as more diagnostic of a person's ‘true nature’, particularly for traits that can be disconfirmed by a single counterexample (Liefgreen et al., 2020; Trafimow & Schneider, 1994). For example, a single act of dishonesty from an otherwise honest person may be especially revealing. Research in judgement and decision‐making shows that people weight information by its diagnostic value rather than simply favouring consistency (e.g. Beyth‐Marom & Fischhoff, 1983; Nelson, 2008; Trope & Bassok, 1982). Because expectation‐violating information is often perceived as highly informative, it tends to carry greater weight in updating impressions. This dynamic can manifest as a contrast bias in impression updating, where the inconsistency creates a sharper divergence from the initial impression (when compared to the impression update under consistent information).

### NATURE OF THE IMPRESSION TARGET (SOCIAL VS NON‐SOCIAL)

Besides the consistency in the valence of new information, does the nature of the impression target matter? Specifically, does it matter whether the impression is about a social target (i.e. a person) or a non‐social target (i.e. a non‐human object)? It has been argued that social targets may trigger associations or emotions that are not triggered by non‐social targets. For example, people may engage in trait inference only for social impression targets, which means that people infer traits from the behaviour of others. These inferred traits then serve as a cognitive filter, influencing how future information about that person is interpreted, weighed or selectively attended to Uleman & Kressel (2013). For non‐social targets, the reference to ‘intentions’ would not be relevant. Additionally, for social targets, negative behaviours are often seen as indicative of the internal characteristics of the individual (Baumeister et al., 2001; Cuddy et al., 2011; Uleman & Kressel, 2013). In contrast, impressions about non‐social objects may only be shaped by observable features. This may result in less bias compared to social targets.

However, opposing arguments highlight key distinctions in how new information is processed when forming impressions on either social or non‐social targets. More precisely, social cognition is not necessarily different from other cognitive processes. For example, the Situated Social Cognition theory (Smith & Semin, 2007) suggests that our cognition functions, including those that do not have an obvious link with social situations, are almost always shaped by social processes. This indicates that our cognitive processes are fundamentally similar regardless of whether the target is social. While we may not attribute intentions or personality traits to non‐social targets, we can still form stable, affect‐laden impressions—for example, by perceiving a house as ‘welcoming’. These impressions may function in the same way as impressions of human characteristics, guiding attention and shaping interpretation of new information. In this way, the processes underlying impression formation and updating may be comparable across social and non‐social domains.

## THE PRESENT RESEARCH

The present work makes both theoretical and methodological contributions. Although confirmation bias and contrast effects driven by information diagnosticity have each been well‐documented, previous work has largely examined them independently or treated them as separate phenomena. We instead conceptualize confirmation and contrast as competing theories of the same impression updating process and test them directly by evaluating their opposing predictions about how impressions evolve as new evidence accumulates.

Our use of the term ‘bias’ differs from classic anchoring‐and‐adjustment accounts (Hogarth & Einhorn, 1992), which are purely descriptive: they parameterize how an impression assimilates incoming evidence but define no normative standard for what updating should be. Within that framework, updating cannot be characterized as biased or unbiased. We instead adopt a Bayesian ideal observer as a normative benchmark and define bias as directional departures from it.

A second question concerns the scope of these dynamics. If updating depends on trait inference specific to persons (Uleman & Kressel, 2013), confirmation and contrast should be more pronounced for social than non‐social targets; if updating instead draws on domain‐general processes (Situated Social Cognition; Smith & Semin, 2007), the same bias structure should hold across target types. Comparing the two therefore tests trait inference against domain‐general accounts.

### METHODOLOGICAL INNOVATION

Beyond these theoretical questions, the present work also introduces two methodological advances. Research on cognitive bias typically relies on single‐session laboratory paradigms. Studies of biased information search (e.g. Kaanders et al., 2022), interpretation (e.g. Taber & Lodge, 2006) and memory (e.g. Vedejová & Čavojová, 2022) often examine bias within a one‐time lab visit. While informative, such short‐term paradigms may limit generalizability to real‐world impression updating, which unfolds through repeated exposure to information over days, weeks or longer. Over extended intervals, confirmation bias may weaken as prior impressions become less salient and motivations for consistency diminish, raising the question of whether confirmation bias persists or attenuates as contradictory information accumulates over time.

To address this gap, we introduce Ecological Momentary Experimentation (EME), an experimental paradigm adapted from Ecological Momentary Assessment (EMA). Unlike traditional EMA, which is observational, EME experimentally presents information at natural intervals (e.g. daily) and records participants' responses over time. This approach allows impressions to form and evolve under controlled yet ecologically valid conditions.

A further methodological contribution is our use of a Bayesian ideal‐observer framework together with group‐level random‐effects model selection. We formalized impression updating within a Bayesian framework and compared alternative bias structures using group‐level random‐effects model selection (Rigoux et al., 2014; Stephan et al., 2009), allowing different participants to be characterized by different updating mechanisms rather than assuming a single model fits all. It also differs from traditional reinforcement learning models of impression updating (e.g. Hackel et al., 2019; Lockwood & Klein‐Flügge, 2021), which typically assume either no prior beliefs or fixed prior weighting. In contrast, our framework explicitly tested whether prior beliefs shape the integration of new information and allowed uncertainty to modulate updating, such that more certain impressions changed less readily.

### RESEARCH QUESTIONS

Using two experiments, we address three research questions. First, what kind of bias is the most dominant in daily exposure to new information while forming impressions? We hypothesized that confirmation bias would dominate impression updating (H1; Nickerson, 1998; Rabin & Schrag, 1999). Second, is there a difference in the magnitude of this bias depending on the valence of the initial information? Although confirmation bias has been shown to operate across both positive and negative initial impressions, negativity‐bias theory suggests that negative information is weighted more heavily than positive information (Baumeister et al., 2001; Rozin & Royzman, 2001). We therefore hypothesized that confirmation bias would be stronger when the initial impression was negative than when it was positive (H2). Third, does the magnitude of this bias differ between social and non‐social targets? Given that confirmation bias in impression formation has been linked to motivational factors such as affiliation goals (Sinclair et al., 2005; Ybarra et al., 2001), we hypothesized that confirmation bias would be stronger for social than for non‐social targets (H3).

## STUDY 1

### Methods

#### Open practices

This research was preregistered (https://osf.io/uaze7) with the prediction that confirmation bias would dominate daily impression updating (H1), that it would be stronger following negative than positive initial impressions (H2), and that it would be stronger for social than non‐social targets (H3). The preregistered analyses focused on comparing variants of confirmation bias models. However, inspection of the raw data revealed patterns inconsistent with these predictions: specifically, ratings on the third day (after equal exposure to positive and negative information) did not conform to confirmation bias. We therefore adopted a more exploratory approach and developed a general modelling framework capable of detecting multiple biases, including positive, negative, confirmation and contrast bias. In addition, impression ratings plateaued during the final days, suggesting limited updating. To account for this pattern, we introduced the Bayesian Triggered Bias Model, which allows updating to occur conditionally on informational inconsistency. These deviations from the preregistered plan were motivated by the goal of aligning computational explanations with the observed data.

All data, analysis code and study materials have been made publicly available at OSF and can be accessed at https://osf.io/yrm4v. Data were analysed using MATLAB R2019a and the offline version of the package VBA toolbox downloaded from https://mbb‐team.github.io/VBA‐toolbox/download‐registered/. Following recommendations by Appelbaum et al. (2018), we report how we determined our sample size, describe all experimental manipulations and disclose all measurements used in this study.

#### Participants

Participants were recruited via Prolific (https://www.prolific.com/) and were UK‐based, fluent in English and had managerial experience. An a priori power analysis based on an effect size from related work on cognitive bias (*d* = 0.28; Menne‐Lothmann et al., 2014) indicated that 100 participants (50 per group) were required to achieve 80% power at *α* = .05 (two‐tailed). Accordingly, 100 participants were recruited. Fourteen were excluded due to technical issues, leaving a final sample of 86 participants (*M*age = 41.35, *SD* = 10.57; 46 men, 40 women). With this sample size, the minimum detectable effect size was approximately *d* = 0.31.

#### Procedure

All study procedures were approved by the Ethics Committee of the Faculty of Social Sciences at Radboud University (ECSW‐2024‐015). Participants provided informed consent and received instructions to install the m‐path app (Mestdagh et al., 2023). Beginning the following day, they completed baseline questions on age, gender and sense of power (the latter was preregistered but is not analysed here, as the observed patterns reflected a different bias).

**FIGURE 1:**
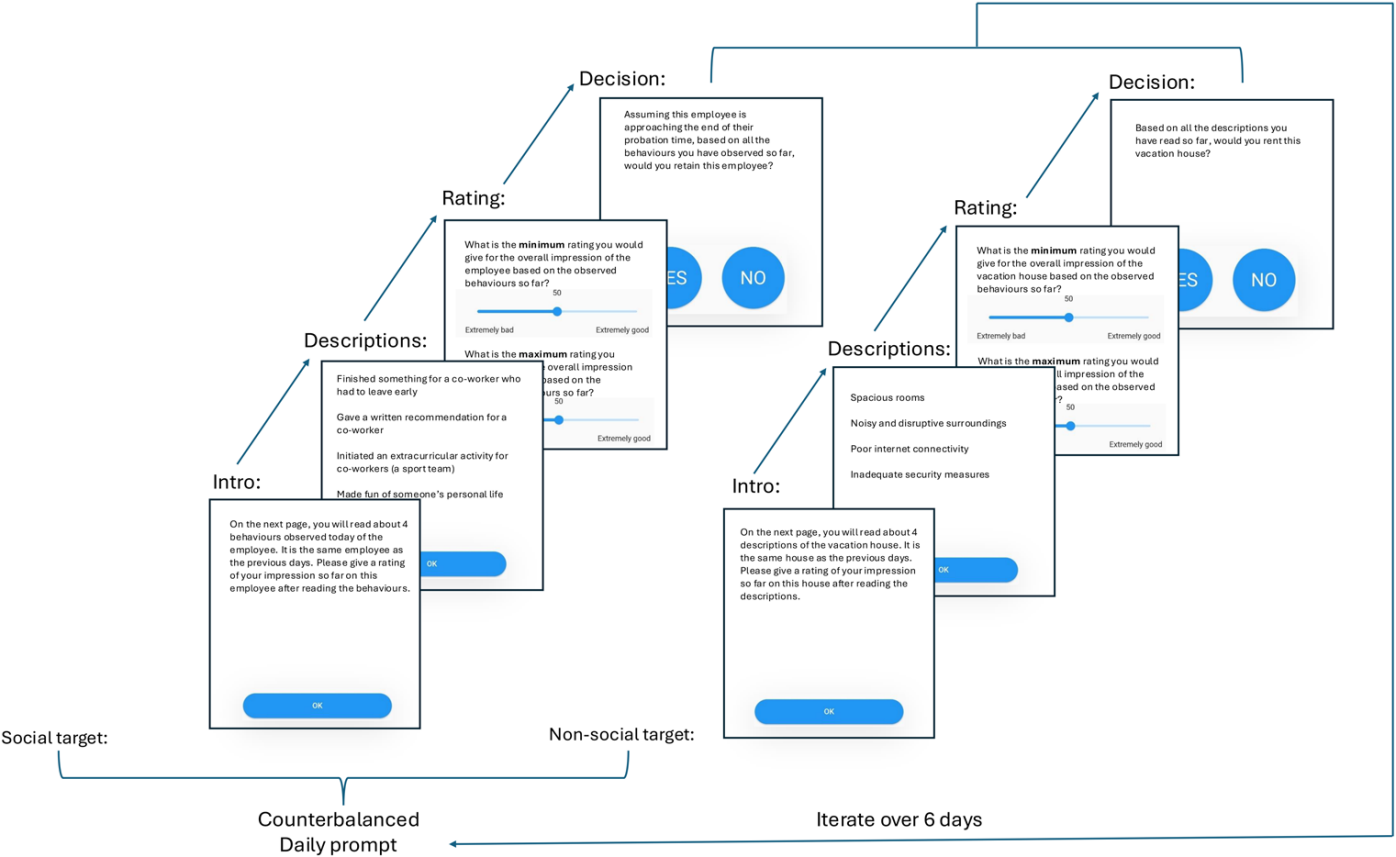
Structure of a daily impression‐updating session. Each day, participants evaluated the social (virtual employee) and non‐social (vacation house) targets in counterbalanced order. For each target, four behavioural descriptions were presented, followed by an impression rating (1–100) and a binary decision. Participants were assigned to one of two conditions differing in whether information became increasingly positive or negative over days. The procedure was repeated for 6 consecutive days.

Participants then completed a 6‐day impression updating task involving two targets: a virtual employee (social) and a vacation house (non‐social). No evaluative information was provided prior to the task; impressions on Day 1 were formed directly from the information presented. Each day, participants received four pieces of information per target and rated their impressions on a 1–100 scale (1 = very bad; 100 = very good), followed by binary hypothetical decisions regarding contract continuation (employee) and rental choice (vacation house). Information for the two targets was presented each day sequentially, with order counterbalanced. Participants could optionally review their previous‐day rating in Study 1 (see Figure 1). Stimulus materials were pretested with an independent sample to verify valence categorization; full pretesting procedure and results are reported in Data S1: Supplementary Material 1.

Participants were randomly assigned to one of two conditions. In Condition 1, information about the employee progressed from predominantly negative to positive, whereas information about the vacation house progressed from positive to negative; Condition 2 followed the opposite pattern. Specifically, on Days 1–3, the ratio of negative to positive information shifted from 3:1 to 1:3, followed by exclusively positive (or negative, respectively) information on Days 4–6 (see Table 1). Behavioural descriptions reflected common workplace and housing attributes (e.g. ‘listened compassionately to a colleague’ vs. ‘refused to help at work’; ‘functional layout’ vs. ‘inefficient design’).

**TABLE 1:** The number of good (G) and bad (B) information sent per day in two conditions (Study 1).

Condition		Day 1	Day 2	Day 3	Day 4	Day 5	Day 6
1	G	3	2	1	0	0	0
	B	1	2	3	4	4	4
2	G	1	2	3	4	4	4
	B	3	2	1	0	0	0

#### Data analysis

The computational models of impression updating. We modelled impression updating using distorted variants of Bayesian belief updating. Let $x\in \left[0,1\right]$ denote a latent trait of the target (social or non‐social), with higher values indicating more positive impressions. Participants' impressions were modelled as probabilistic beliefs about x.

As a normative benchmark, we implemented a Baseline Bayesian Model, in which beliefs are updated by integrating observed positive (G) and negative (B) information over time:

(1) $$P\left(x|G_{1}\ldots G_{i+1},B_{1}\ldots B_{i+1}\right)=\text{Beta}\left(a_{i+1},b_{i+1}\right),=\text{Beta}\left(a_{i}+G_{+1},b_{i}+B_{i+1}\right)$$

This model represents an ideal Bayesian observer who weights positive and negative information equally; the expected impression corresponds to the proportion of positive information observed so far, with certainty increasing as more information accumulates (see Data S1: Supplemental Material 2 for details).

To capture biased updating, we extended this model by introducing a bias parameter θ that asymmetrically weights positive and negative information:
(2)

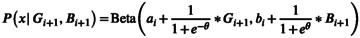




We refer this model as the Bayesian Bias Model. In this model, θ determines whether positive information is overweighted (positive bias), underweighted (negative bias) or weighted equally (no bias). Patterns of 𝜃 across experimental conditions distinguish confirmation bias from contrast bias. Specifically, confirmation bias is inferred when information congruent with the initial impression is weighted more strongly, whereas contrast bias is inferred when information incongruent with the initial impression receives greater weight.

Because impression ratings plateaued during the final days of the task, we tested a Bayesian Triggered Bias Model, in which impressions are updated only when new information meaningfully changes the balance between positive and negative input. When the informational balance remains unchanged, impressions remain stable:
(3)

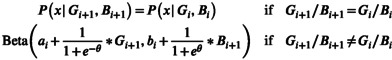




This model simply added one more constraint: That the update only happens if the ratio of the positive and negative information changes compared to the previous day. For example, when participants receive two pieces of positive information and one piece of negative information 1 day and four pieces of positive information and two pieces of negative information the next day, they do not update their impressions; however, if they receive three pieces of positive information and two pieces of negative information, they will update their impressions. This simple extension of the Bayesian Bias Model captures the observed plateau in the last 3 days.

To test the necessity of priors, we additionally tested a variant of the bias model (the Cumulative Triggered Bias Model) that excludes a prior and updates impressions only when the ratio of positive to negative information changes:

(4) $$\begin{array}{ccc} x_{i}=x_{i-1} & \text{if} & G_{i+1}/B_{i+1}=G_{i}/B_{i}\\ \frac{G_{1:i}*\frac{1}{1+e^{\theta }}\;}{G_{1:i}*\frac{1}{1+e^{\theta }}+B_{1:i}*\frac{1}{1+e^{-\theta }}} & \text{if} & G_{i+1}/B_{i+1}\neq G_{i}/B_{i} \end{array}$$

Additionally, we tested models that assume that participants rely on a sliding memory window of fixed capacity, ranging from 1 to 6 days (Cumulative Bias Models). In these models, impressions are formed based on the most recent M days, by weighting positive and negative outcomes asymmetrically:
(5)

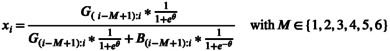




Here we define *M* as the width of the sliding memory window, $G_{\left(t-M+1\right):t}$ as the cumulative positive information from Day *i*−*M + 1* to Day *i* or from Day 1 to Day t if i‐*M* + 1 < 1. Similarly, $B_{\left(i-M+1\right):t}$ denotes the cumulative negative information over the same interval.

#### Model fitting and comparison

We used the Variational Bayesian Analysis (VBA) toolbox to fit model parameters (*θ*) to each participant's impression ratings over time, separately for each condition. All candidate models were fit at the individual level, yielding parameter estimates and model evidence that accounts for both goodness of fit and model complexity, enabling formal model comparison (Daunizeau, 2018; Daunizeau et al., 2014).

We then compared group‐level model evidence across the Baseline Bayesian Model, Bayesian Bias Model, Bayesian Triggered Bias Model, Cumulative Bias Model and Cumulative Triggered Model, grouping model variants where applicable. Model comparison was performed using random‐effects Bayesian model selection, summarized by exceedance probability (EP) and protected exceedance probability (PEP), which estimates the likelihood that a given model best explains the data, with PEP providing a more conservative adjustment for chance (Rigoux et al., 2014; Stephan et al., 2009).

#### Parameter analysis

Subsequently, we conducted a model comparison among the three models. If the Bayesian Bias Model or the Bayesian Triggered Bias Model was selected, we examined the estimated parameters to determine the most dominant bias (Research Question 1). Additionally, we performed *t*‐tests to compare the means of the extracted parameters across conditions, addressing Research Questions 2 and 3. If the Baseline Model was chosen, it indicated that no bias was present in the data. However, if EP or PEP suggested no clear superior model, we applied Bayesian Model Averaging (i.e. weighting parameters by each model's evidence) to calculate an integrated parameter for the magnitude of the bias. These integrated parameters were then compared across conditions using *t*‐tests.

### Results

**FIGURE 2:**
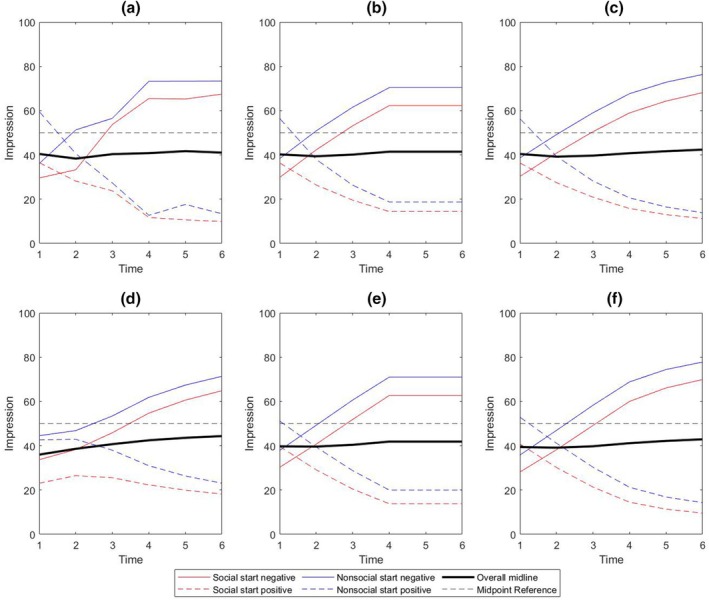
Study 1 Model Fit Visualization: Panel (a) displays the trends in the raw data (averaged across all participants). Panel (b) presents the predicted values from the Bayesian Triggered Bias Model, Panel (c) shows the predicted values from the Bayesian Bias Model, Panel (d) depicts the predicted values from the Baseline Bayesian Model. Panel (e) shows the predicted values from the Cumulative Triggered Bias Model, which is a variant of Panel (b) Bayesian Triggered Bias Model without a prior. Panel (f) represents the Cumulative Bayesian Bias Model, which is the Bayesian Bias Model without a prior.

The model comparison indicated that the Cumulative Triggered Model was overwhelmingly favoured (EP ≈ 1.00, PEP ≈ 1.00). As shown in Figure 2, its predictions closely match those of the Bayesian Triggered Bias Model, which incorporates a prior. However, the Cumulative Triggered Model was preferred due to its simpler structure and comparable results.

The means of integrated parameters extracted from the models are shown in Table 2. Negative bias (parameters below 0: (*t*(85) = 11.09, 95% CI [1.47, 2.12], *p <* .001, Cohen's *d* = 1.20)) was observed in the condition with a positive start, while positive bias (parameters above 0: (*t*(85) = 2.00, 95% CI [0.00, 0.44], *p* = .049, Cohen's *d* = 0.22)) was observed in the condition with a negative start. In other words, people exhibit a significant valence bias, whose sign depends upon the condition (i.e. whether information starts positive or negative). More precisely, the sign of the valence bias is opposite to that of the first piece of information, indicating that people exhibit a contrast bias (Research Question 1).

**TABLE 2:** Parameters (θ) per cell extracted from the Cumulative Triggered Model (Mean ± SE).

	Social	Non‐social
Start positive	−2.50 ± 0.27	−1.16 ± 0.13
Start negative	−0.04 ± 0.18	0.49 ± 0.10

The magnitude of the bias (in absolute values) was stronger (*t*(170) = 6.30, *p <* .001, 95% CI [0.77, 1.47], Cohen's *d* = 0.96) when the starting point was positive (*M* = 1.82, *SD* = 1.47) compared to negative (*M* = 0.70, *SD* = 0.76) (Research Question 2). The results showed a difference in the magnitude of the bias (*t*(170) = 3.47, 95% CI [0.36, 1.31], *p* = <.001, Cohen's *𝑑* = 0.53) between social condition (*M* = 1. 59, *SD* = 1.62) compared to non‐social one (*M* = 0.94, *SD* = 0.72) (Research Question 3). However, this result should be interpreted with caution because the study design had some flaws. As shown in Figure 2, the ratings in the ‘Social start positive’ condition did not start above 50, which means that the initial stimulus on the first day was somewhat negative. This may have influenced the parameter estimates, particularly since this imbalance was not compensated for in the model.

Decisions tracked impression ratings closely across days, consistent with the impression‐updating dynamics described above. A formal analysis of the impression–decision relationship for both studies is reported in Data S1: Supplemental Material 3.

## STUDY 2

Study 1 revealed differences in bias magnitude and valence between positive and negative starting points and between social and non‐social targets. However, because initial impression ratings did not fully reflect the intended starting valence (most notably in the ‘Social start positive’ condition) some of the observed differences may reflect anchoring on these initial ratings rather than genuine differences in impression‐updating bias. Besides, it was not clear whether the plateau at the last few days was because of information saturation, or lack of trigger for people to update their impression. To address this uncertainty and eliminate alternative explanations, Study 2 incorporated design improvements focused on clarifying the initial impression, ruling out the information saturation explanation and eliminating potential order or anchor effects.

### Methods

#### Participants

For the second study, we aimed to replicate and extend the findings of Study 1 using an improved design. Rather than basing the sample size on a new power analysis, we used the same target sample size as in Study 1 to ensure consistency across studies and sufficient power to detect effects of comparable magnitude. In total, 97 participants were recruited through Prolific. All participants were located in the United Kingdom and had prior managerial experience. Among them (*M*_age_ = 38.65, *SD*_age_ = 10.62), there were 59 females and 38 males. With this sample size (*N* = 97), the minimum detectable effect size (Cohen's *d*) was approximately 0.29 at *α* = .05 (two‐tailed) and 80% power, indicating the analysis was sensitive to small‐to‐medium effects.

#### Procedure

The procedure of Study 2 was largely the same as in Study 1 but with a few key differences:

1. In Study 2, the information sent on the first 2 days was overwhelmingly positive or negative in each condition. The sequence of behaviours is shown in Table 3. In this design, the sixth day is when participants encounter an equal number of positive and negative pieces of information. This ensures that the initial impression starts at the end of the spectrum.
2. As can be seen in Table 3, the ratio of positive and negative information remained the same across the first 2 days. This eliminates the possibility that participants give the same rating for these days simply because they are already certain of their impression, assuming this pattern of impression ratings occurs.
3. Within each day, the order of positive and negative behaviours was randomized. In addition, the assignment of specific behaviours to each day was randomized across the 6‐day period, ensuring that no fixed sequence of individual items was consistently presented in the same temporal position. This design reduces the likelihood that findings are driven by order effects or systematic primacy of particular behaviours rather than their evaluative content.
4. In Study 2, participants were not given the option to review their rating from the previous day. This was done to prevent anchoring effects, whereby participants might base their new ratings on their previous ones rather than their current impressions.

**TABLE 3:** The number of good (G) and bad (B) information sent per day in two conditions (Study 2).

Condition		Day 1	Day 2	Day 3	Day 4	Day 5	Day 6
1	G	3	3	2	1	0	0
	B	0	0	1	2	3	3
2	G	0	0	1	2	3	3
	B	3	3	2	1	0	0

### Results

**FIGURE 3:**
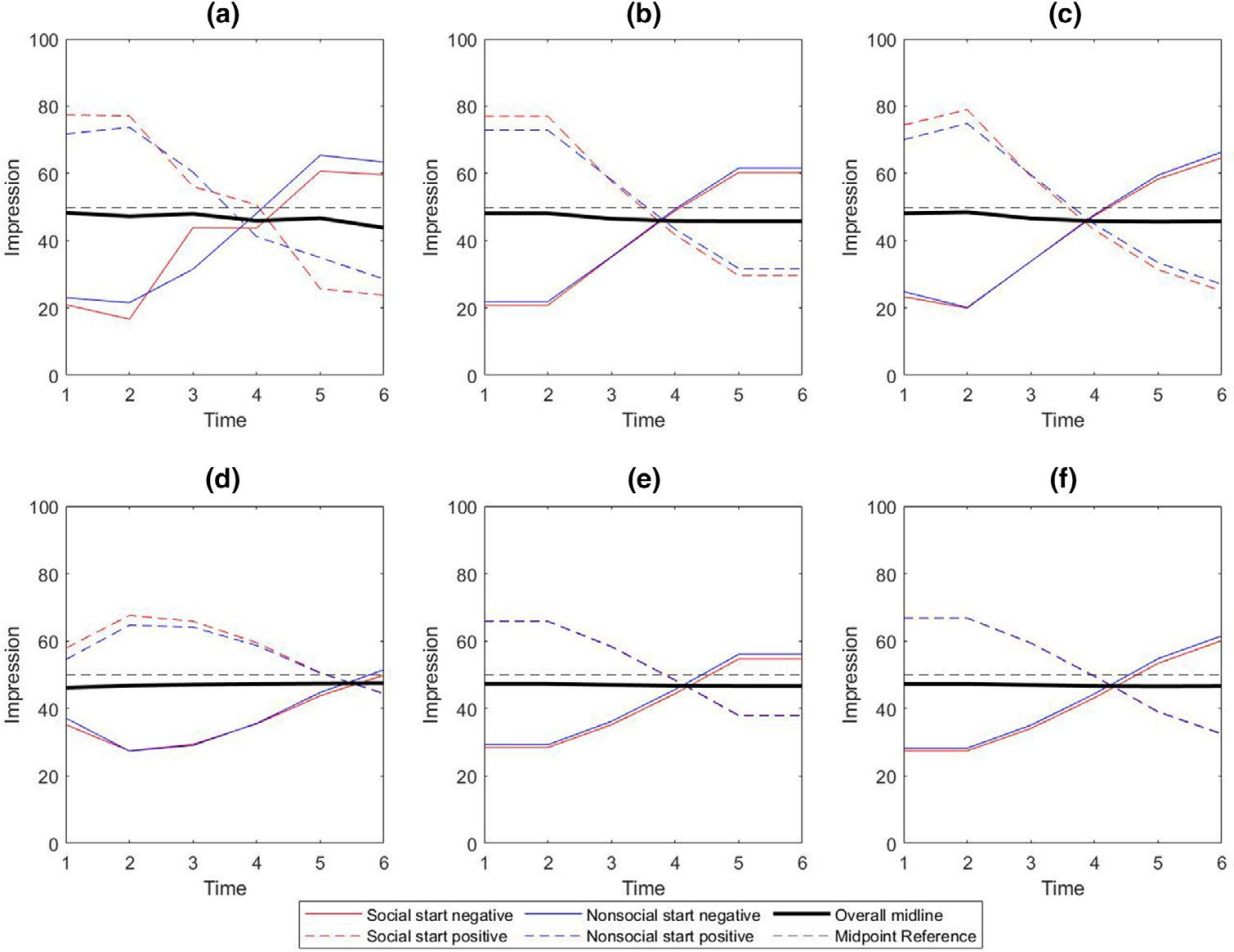
Study 2 Model Fit Visualization (selected models): Panel (a) displays the trends in the raw data (averaged across all participants). Panel (b) presents the predicted values from the Bayesian Triggered Bias Model, Panel (c) shows the predicted values from the Bayesian Bias Model and Panel (d) depicts the predicted values from the Bayesian Baseline Model. Panel (e) shows the predicted values from the Cumulative Triggered Bias Model, which is a variant of Panel (b) Bayesian Triggered Bias model without a prior. Panel (f) represents the Cumulative Bayesian Bias Model, which is the Bayesian Bias Model without a prior.

The visualization of raw data compared to the model predictions is shown in Figure 3.

The model comparison indicated that the Cumulative Triggered Model was overwhelmingly favoured (EP ≈ 1.00, PEP ≈ 1.00). This indicates that the Cumulative Triggered Bias Model overwhelmingly outperformed the other models. Similar to Study 1, although the Bayesian Triggered Bias Model (Panel b in Figure 3) appear closer to the raw data, the model selection indicated that a parsimonious mechanism (i.e., no priors) explains the majority of the dynamics. The similarity between Panel b and the raw data could have been due to overfitting. In other words, people in general do not have priors when forming impressions, but they do show a valance bias in impression updating, and the updating happens only when the ratio of positivity and negativity changes. This result is consistent with Study 1, indicating an internal replication between the two studies.

We then tested whether the valence bias was dependent upon the condition. As for Study 1, we relied upon standard statistical significance testing on estimated model parameters extracted from the chosen model (see Table 4).

**TABLE 4:** Parameters (θ) per cell extracted from the Cumulative Triggered Bias Model (Mean ± SE).

	Social	Non‐social
Start positive	−1.36 ± 1.68	−1.57 ± 1.21
Start negative	0.87 ± 1.61	0.95 ± 1.01

The results confirmed that participants showed positive bias (*t*(96) = 6.72, 95% CI [0.64, 1.18], *p* < .001, Cohen's *d* = 0.68) when their initial impression was negative, and negative bias (*t*(96) = 9.88, 95% CI [1.17, 1.76], *p* < .001, Cohen's *d* = 1.00) when their initial impression was positive. This replicates the findings of contrast bias in Study 1 for Research Question 1. The magnitude (in absolute values) of the negative bias after a positive start (*M* = 1.23, *SD* = 1.04) was significantly bigger (*t*(192) = 2.26, 95% CI [0.05, 0.71], *p* = .025, Cohen's *d* = 0.33) than the magnitude of the positive bias after a negative start (*M* = 1.08, *SD* = 1.30) (Research Question 2). This result was consistent with Study 1.

There was no significant difference (*t*(192) = 0.15, 95% CI [−0.41, 0.26], *p* = .884, Cohen's *d* = 0.02) between the social (*M* = −0.26, *SD* = 1.98) and non‐social (*M* = −0.21, *SD* = 2.37) target (Research Question 3). After refining the design in Study 2, the previously observed difference between social and non‐social impressions was no longer significant.

Consistent with Study 1, the decisions again tracked impression ratings closely (see Data S1: Supplemental Material 3).

## DISCUSSION

Across two studies, we aimed to investigate the valence and magnitude of biases in impression updating. We used an experimental design called the Ecological Momentary Experiment, which provided a more ecologically valid approach to assessing real‐world impression updating and decision‐making processes. Furthermore, we used computational modelling to identify hidden bias mechanisms that impact peoples' impression updating.

Contrary to our preregistered prediction that confirmation bias would dominate (H1), our findings reveal a clear contrast bias in impression updating (research question 1). The contrast bias magnitude was stronger when the impression changed from positive to negative (research question 2), consistent with the negativity asymmetry predicted by H2. However, we found no consistent evidence for a difference between the impression‐updating process of social and non‐social targets (research question 3), contrary to the social amplification predicted by H3.

Building on extensive research on confirmation bias and assimilative updating (e.g. Nickerson, 1998; Vedejová & Čavojová, 2022), the present study provides a novel test of contrast bias in sequential impression updating about the same target over time. While prior research has documented contrast effects in comparative or one‐shot judgement contexts, our results show how contrast bias emerges dynamically over time as impressions are repeatedly updated in response to inconsistent information.

### Contrast bias, conditional updating and valence asymmetry

We found evidence for contrast bias in sequential impression updating. Specifically, when the initial impression was negative, new positive information had a greater impact on changing the impression. Similarly, when the initial impression was positive, new negative information had a greater impact on updating the impression. It seems that participants were engaging in the comparison between the information presented on each day to the information presented on the previous day. As a result, the change in the ratio became salient. Importantly, this process is different from the recency effect, where greater emphasis is placed on the most recent input. In our design, a recency effect would predict that both the negative and positive parts of the new information receive more weight. We ruled out this alternative explanation by fitting a recency effect model. According to random‐effect Bayesian model selection, this model proved to be a much less likely explanation for our data (see Data S1: Supplemental Material 4). While contrast biases are well‐documented in areas such as visual perception (e.g. Mach, 1965), facial attractiveness (e.g. Lei et al., 2020), consumer choice (e.g. Voichek & Novemsky, 2024) and education (e.g. Yeates et al., 2015), the application in impression updating had received comparatively less attention. Previous research on social cognition has addressed related concepts in shaping changes in impression, such as the role of expectancy violation, diagnostics and surprise (e.g. Fiske & Neuberg, 1990; Van Overwalle & Labiouse, 2004). The current findings build on this foundation and offer a formalized, dynamic explanation for contrast effects in daily updating of impressions using computational modelling.

The occurrence of the contrast bias can be explained by the surprise‐attention link identified in previous literature (for a review, please see Horstmann, 2015), which suggests that surprising or novel stimuli naturally capture attention and sustain engagement. This heightened attention to unexpected information likely amplifies its influence on impression updating, leading to the observed contrast bias. In our study, contradictory information may have appeared novel, surprising and salient, leading participants to overweight it. Research has shown that surprising and salient information readily captures our attention (Itti & Baldi, 2009; Van Overwalle & Labiouse, 2004). Consequently, surprising events often dominate our perceptions and memories. In a similar vein, inconsistent information is often perceived as more telling than consistent information. This expectation‐violation framework may also be relevant for understanding the valence asymmetry in contrast effects, as discussed further below. Consistent with this view, Trope and Bassok (1982) argued that perceivers preferentially value diagnostic information—information that best discriminates between competing hypotheses—over information that merely confirms an existing expectation. In Bayesian terms, a piece of information carries more weight when it clearly favours one possibility over another (Ajzen & Fishbein, 1975; Beyth‐Marom & Fischhoff, 1983). In our study, surprising information (behaviours that contradict the initial impression) plays exactly this role, which is why it is weighted more heavily in updating.

The longer interval in the current study may also explain the contrast bias observed here, rather than the confirmation bias more commonly reported in impression research (e.g. Nickerson, 1998; Vedejová & Čavojová, 2022). Over short intervals, people may maintain consistent impressions to avoid cognitive dissonance (Bhadauria et al., 2024), leading to a tendency for confirmation bias. Over longer intervals, however, participants may become less attached to prior judgements and focus more on changes in incoming information, leading to stronger updating. It is worth noting that, although larger shifts in impressions were observed under these conditions, updating still reflected integration of the full history of prior information. Thus, the observed pattern can be understood as resulting from cumulative, history‐dependent belief updating, in which new evidence is evaluated relative to previously encountered information rather than in isolation.

The findings suggest that participants did not rely on explicit prior information when updating their impressions, as including a prior term in the Bayesian models did not improve model fit. Instead, impression updating appeared to rely primarily on bottom‐up processing, consistent with previous research showing that bottom‐up information is favoured when evaluating unfamiliar or uncategorized targets (Wood & Hutter, 2011). Because participants received minimal information about the targets and were not given bias‐inducing details (e.g. gender or prior stories), pre‐existing expectations were likely limited. Studies that manipulate participants' prior expectations may therefore yield different results.

The finding that impressions update only when there is a change in the ratio of positive to negative information suggests that consistent and inconsistent information are processed differently, with consistent information often exerting less influence on impression updating. When there is a noticeable and meaningful difference or change, this change will be exaggerated as a result of the contrast bias (Mende‐Siedlecki & Todorov, 2016). When the new information is consistent with previous information, it does not trigger significant cognitive engagement or reevaluation. What happens is possibly a reinforcement of the impression in terms of certainty, but not reflected in the value of the rating. Alternatively, it is also possible that no cognitive updating occurs at all, leaving both the certainty and the rating unchanged. Since processing inconsistent information requires more cognitive effort (Mende‐Siedlecki et al., 2013), updating impressions only when inconsistencies arise may serve as a strategy for managing cognitive load. In other words, prioritizing the most diagnostic information may help conserve cognitive resources.

The valence asymmetry partly aligns with the general findings of negative bias in information processing (Fan et al., 2023; Ito et al., 1998; Torres et al., 2024). Negative experiences are often associated with stronger emotional responses, which makes them more memorable and has a bigger impact on the impression (Kensinger, 2009; Williamson et al., 2019). Besides, the stronger negative contrast (than the positive contrast) is possibly due to the fact that a person who frequently exhibits bad behaviour cannot do so constantly and will also display good behaviour, but a person showing good behaviour is not expected to show any bad behaviour (Cuddy et al., 2011). As our negative behaviours were predominantly moral transgressions (from a CWB scale), this stronger negative contrast is consistent with the greater diagnosticity of negative information in the morality domain; competence behaviours might show the reverse (Skowronski & Carlston, 1989). More broadly, behaviour violating stronger expectations produces stronger dispositional inferences. Classic attribution research showed that unexpected behaviour is more informative about underlying traits, because behaviour consistent with social norms or role demands can be attributed to situational pressure, whereas behaviour that deviates from such expectations has fewer alternative explanations and is therefore more readily attributed to the actor's disposition (Jones & Davis, 1965), and a similar pattern has been documented in persuasion research, where communicators advocating unexpected positions carry greater influence because they cannot be easily attributed to the communicator's bias and are therefore seen as more reflective of reality (Eagly et al., 1978). Because positive behaviour is generally expected, negative behaviour/information violates a stronger baseline expectation and is therefore weighted more heavily in impression updating. Therefore, negative behaviours are often seen as more indicative than positive behaviours (Cuddy et al., 2011; Uleman & Kressel, 2013).

### No consistent evidence for a difference between social and non‐social impression targets

We found no consistent evidence that bias in impression updating differed between social and non‐social targets. Although Study 1 suggested a difference, Study 2 showed this was likely due to a design limitation: the supposedly positive‐start social condition did not receive a positive average initial rating. After correcting this issue, the difference disappeared. This finding is consistent with theories proposing that social and non‐social processing are not fundamentally distinct (Gross & Medina‐DeVilliers, 2020; Smith & Semin, 2007). In particular, the idea that people can correct mistakes whereas flaws in non‐social targets are permanent did not appear to influence updating. Although increasing positive behaviours could signal corrective action, and increasing negative behaviours could reveal true character (Cuddy et al., 2011; Mende‐Siedlecki et al., 2013), participants appeared to rely mainly on the immediate balance of positive and negative information. This may reflect the study design, which emphasized behavioural valence rather than encouraging inferences about traits or intentions. Because the behaviours could reflect different dimensions such as competence, warmth, honesty or reliability, the design may have limited multidimensional trait inference (Uleman & Kressel, 2013). Nevertheless, since real‐world decisions often reduce impressions to overall valence, the similar updating patterns across social and non‐social targets may indicate reliance on similar heuristic evaluation strategies.

Another possible explanation for this finding is the absence of a clearly defined evaluative goal. Recent work on diagnosticity suggests that the integration of information depends on contextual factors such as the hypothesis being tested (e.g. whether a job requires agency or communication skills) and base‐rate expectations about the target group (e.g. whether a typical group member is expected to be positive or negative) (Ziegler et al., 2026). In our study, participants were not provided with a concrete evaluative hypothesis (e.g. suitability for a specific role or a particular vacation goal), nor were they given group‐based expectations about the target. As a result, participants may have relied on more general evaluation strategies, which could be similar across both social and non‐social targets.

### Limitations and future directions

Before closing, there are a few limitations to consider. First, the model used counts of positive and negative behaviours with a global weighting parameter, capturing overall learning dynamics without distinguishing between item‐level differences in diagnosticity or extremity. Because more extreme behaviours are often perceived as more informative (Skowronski & Carlston, 1987), Study 2 randomized information order to reduce potential sequence effects driven by particularly salient items. Nevertheless, behaviours of the same valence were still treated equivalently at the input level, and future work could build on the current approach by incorporating subjective diagnosticity or extremity ratings to allow more fine‐grained evidence integration.

Second, although the use of virtual employees and vacation houses provided strong experimental control, it may limit generalizability. Real‐world impression updating often involves active information sampling and interactive information flow (Denrell, 2005), both of which were absent in the present paradigm. At the same time, experience‐sampling research suggests that contextual factors such as mood or environment may play a smaller role in trait impressions than stable perceiver and target characteristics (Xie et al., 2023). Nevertheless, it remains unclear whether the contrast bias and valence asymmetries observed here extend to real interpersonal interactions, consequential decisions or more diverse target categories. Future research should examine the robustness of these effects across broader contexts.

Third, the study duration of 6 days was a manageable timeframe for data collection, but this short duration restricted our ability to test more complex models or capture longer‐term changes such as habituation or belief consolidation. Future research could incorporate longer study periods with continuous sampling to examine how daily impression‐updating dynamics evolve over extended timescales. Finally, our paradigm measured explicit impression ratings; whether similar dynamics would emerge in implicit measures of impression updating remains an open question (Ferguson et al., 2019), which future work could address by incorporating implicit measures alongside explicit ratings.

## CONCLUSION

In conclusion, we found evidence for contrast bias in sequential impression updating over time, particularly when impressions shifted from positive to negative, while observing no consistent differences between social and non‐social targets. Using an ecological momentary experimentation paradigm combined with computational modelling, we were able to capture these updating dynamics under controlled yet temporally extended conditions. By examining how impressions evolve within individuals across repeated exposures, the present work extends existing research on impression updating from static demonstrations to dynamic, longitudinal processes. More broadly, these findings highlight the importance of studying impression updating as a temporally extended process, in which early impressions shape how subsequent information is integrated.

## AUTHOR CONTRIBUTIONS

**Fred Hasselman:** Conceptualization; methodology; validation; supervision; writing – review and editing. **Jean Daunizeau:** Conceptualization; methodology; validation; supervision; visualization; writing – review and editing. **Johan Karremans:** Conceptualization; supervision; writing – review and editing. **Sabine Geurts:** Conceptualization; supervision; writing – review and editing. **Yingjie Li:** Conceptualization; methodology; data curation; investigation; formal analysis; visualization; project administration; writing – original draft; writing – review and editing.

## CONFLICT OF INTEREST STATEMENT

The authors declare no conflict of interest.

## Supporting information


Data S1.

## Data Availability

All data, analysis scripts, materials and preregistrations for this study are publicly available at the Open Science Framework: https://osf.io/yrm4v/.

## References

[bjso70123-bib-0001] Ajzen, I. , & Fishbein, M. (1975). A Bayesian analysis of attribution processes. Psychological Bulletin, 82(2), 261–277. 10.1037/h0076477

[bjso70123-bib-0002] Amodio, D. M. (2025). A learning and memory account of impression formation and updating. Nature Reviews Psychology, 4(6), 417–432. 10.1038/s44159-025-00445-x

[bjso70123-bib-0003] Antony, J. W. , Van Dam, J. , Massey, J. R. , Barnett, A. J. , & Bennion, K. A. (2023). Long‐term, multi‐event surprise correlates with enhanced autobiographical memory. Nature Human Behaviour, 7(12), 2152–2168. 10.1038/s41562-023-01631-8 37322234

[bjso70123-bib-0004] Appelbaum, M. , Cooper, H. , Kline, R. B. , Mayo‐Wilson, E. , Nezu, A. M. , & Rao, S. M. (2018). Journal article reporting standards for quantitative research in psychology: The APA publications and communications board task force report. American Psychologist, 73(1), 3–25. 10.1037/amp0000191 29345484

[bjso70123-bib-0005] Baumeister, R. F. , Bratslavsky, E. , Finkenauer, C. , & Vohs, K. D. (2001). Bad is stronger than good. Review of General Psychology, 5(4), 323–370.

[bjso70123-bib-0006] Beyth‐Marom, R. , & Fischhoff, B. (1983). Diagnosticity and pseudodiagnosticity. Journal of Personality and Social Psychology, 45(6), 1185–1195. 10.1037/0022-3514.45.6.1185

[bjso70123-bib-0007] Bhadauria, K. , Bisht, P. , Pandey, A. K. , Sharma, M. , & Singh, D. C. S. (2024). Impact of confirmation bias on decision‐making with a moderating role of tolerance for disagreement. Educational Administration Theory and Practices, 45, 2810. 10.53555/kuey.v30i4.2810

[bjso70123-bib-0008] Bless, H. , & Schwarz, N. (2010). Mental construal and the emergence of assimilation and contrast effects: The inclusion/exclusion model. In Advances in experimental social psychology (Vol. 42, pp. 319–373). Academic Press. 10.1016/S0065-2601(10)42006-7

[bjso70123-bib-0009] Cuddy, A. J. C. , Glick, P. , & Beninger, A. (2011). The dynamics of warmth and competence judgments, and their outcomes in organizations. Research in Organizational Behavior, 31, 73–98. 10.1016/j.riob.2011.10.004

[bjso70123-bib-0010] Daunizeau, J. (2018). The variational Laplace approach to approximate Bayesian inference. arXiv. http://arxiv.org/abs/1703.02089

[bjso70123-bib-0011] Daunizeau, J. , Adam, V. , & Rigoux, L. (2014). VBA: A probabilistic treatment of nonlinear models for neurobiological and Behavioural data. PLoS Computational Biology, 10(1), e1003441. 10.1371/journal.pcbi.1003441 24465198 PMC3900378

[bjso70123-bib-0012] Denrell, J. (2005). Why Most people disapprove of me: Experience sampling in impression formation. Psychological Review, 112(4), 951–978. 10.1037/0033-295X.112.4.951 16262475

[bjso70123-bib-0013] Downey, J. , & Christensen, L. (2006). Belief persistence in impression formation. North American Journal of Psychology, 8(3), 479–487.

[bjso70123-bib-0014] Eagly, A. H. , Wood, W. , & Chaiken, S. (1978). Causal inferences about communicators and their effect on opinion change. Journal of Personality and Social Psychology, 36(4), 424–435. 10.1037/0022-3514.36.4.424

[bjso70123-bib-0015] Fan, X. , Xu, Q. , Liu, J. , Xing, H. , Ning, L. , Chen, Q. , & Yang, Y. (2023). The early negative bias of social semantics: Evidence from behavioral and ERP studies. BMC Psychology, 11(1), 249. 10.1186/s40359-023-01286-0 37633981 PMC10464141

[bjso70123-bib-0016] Ferguson, M. J. , Mann, T. C. , Cone, J. , & Shen, X. (2019). When and how implicit first impressions can Be updated. Current Directions in Psychological Science, 28(4), 331–336. 10.1177/0963721419835206

[bjso70123-bib-0017] Fiske, S. T. , & Neuberg, S. L. (1990). A continuum of impression formation, from category‐based to individuating processes: Influences of information and motivation on attention and interpretation. In M. P. Zanna (Ed.), Advances in experimental social psychology (Vol. 23, pp. 1–74). Academic Press. 10.1016/S0065-2601(08)60317-2

[bjso70123-bib-0018] Furnham, A. , & Boo, H. C. (2011). A literature review of the anchoring effect. The Journal of Socio‐Economics, 40(1), 35–42. 10.1016/j.socec.2010.10.008

[bjso70123-bib-0019] Gershman, S. J. , & Cikara, M. (2023). Structure learning principles of stereotype change. Psychonomic Bulletin & Review, 30(4), 1273–1293. 10.3758/s13423-023-02252-y 36973602

[bjso70123-bib-0020] Gross, E. B. , & Medina‐DeVilliers, S. E. (2020). Cognitive processes unfold in a social context: A review and extension of social baseline theory. Frontiers in Psychology, 11, 378. 10.3389/fpsyg.2020.00378 32210891 PMC7076273

[bjso70123-bib-0021] Hackel, L. M. , Berg, J. J. , Lindström, B. R. , & Amodio, D. M. (2019). Model‐based and model‐free social cognition: Investigating the role of habit in social attitude formation and choice. Frontiers in Psychology, 10, 2592. 10.3389/fpsyg.2019.02592 31824378 PMC6881302

[bjso70123-bib-0022] Hahn, U. , & Harris, A. J. L. (2014). What does it mean to be biased: Motivated reasoning and rationality. In Psychology of learning and motivation (Vol. 61, pp. 41–102). Academic Press. 10.1016/B978-0-12-800283-4.00002-2

[bjso70123-bib-0073] Hogarth, R. M. , & Einhorn, H. J. (1992). Order effects in belief updating: The belief‐adjustment model. Cognitive Psychology, 24(1), 1–55. 10.1016/0010-0285(92)90002-J

[bjso70123-bib-0023] Horstmann, G. (2015). The surprise–attention link: A review. Annals of the New York Academy of Sciences, 1339(1), 106–115. 10.1111/nyas.12679 25682693

[bjso70123-bib-0024] Ito, T. A. , Larsen, J. T. , Smith, N. K. , & Cacioppo, J. T. (1998). Negative information weighs more heavily on the brain: The negativity bias in evaluative categorizations. Journal of Personality and Social Psychology, 75(4), 887–900. 10.1037/0022-3514.75.4.887 9825526

[bjso70123-bib-0025] Itti, L. , & Baldi, P. (2009). Bayesian surprise attracts human attention. Vision Research, 49(10), 1295–1306. 10.1016/j.visres.2008.09.007 18834898 PMC2782645

[bjso70123-bib-0026] Jones, E. E. , & Davis, K. E. (1965). From acts to dispositions the attribution process in person perception. In Advances in experimental social psychology (Vol. 2, pp. 219–266). Academic Press. 10.1016/S0065-2601(08)60107-0

[bjso70123-bib-0027] Kaanders, P. , Sepulveda, P. , Folke, T. , Ortoleva, P. , & De Martino, B. (2022). Humans actively sample evidence to support prior beliefs. eLife, 11, e71768. 10.7554/eLife.71768 35404234 PMC9038198

[bjso70123-bib-0028] Kensinger, E. A. (2009). Remembering the details: Effects of emotion. Emotion Review, 1(2), 99–113. 10.1177/1754073908100432 19421427 PMC2676782

[bjso70123-bib-0029] Klayman, J. (1995). Varieties of Confirmation Bias. Psychology of Learning and Motivation, 32, 385–418. 10.1016/S0079-7421(08)60315-1

[bjso70123-bib-0030] Lei, Y. , He, X. , Zhao, T. , & Tian, Z. (2020). Contrast effect of facial attractiveness in groups. Frontiers in Psychology, 11, 2258. 10.3389/fpsyg.2020.02258 33041899 PMC7523431

[bjso70123-bib-0031] Liefgreen, A. , Pilditch, T. , & Lagnado, D. (2020). Strategies for selecting and evaluating information. Cognitive Psychology, 123, 101332. 10.1016/j.cogpsych.2020.101332 32977167

[bjso70123-bib-0032] Lockwood, P. L. , & Klein‐Flügge, M. C. (2021). Computational modelling of social cognition and behaviour‐a reinforcement learning primer. Social Cognitive and Affective Neuroscience, 16(8), 761–771. 10.1093/scan/nsaa040 32232358 PMC8343561

[bjso70123-bib-0033] McKenzie, C. R. M. (2004). Hypothesis testing and evaluation. In Blackwell handbook of judgment and decision making (pp. 200–219). John Wiley & Sons, Ltd. 10.1002/9780470752937.ch10

[bjso70123-bib-0034] Mende‐Siedlecki, P. , Cai, Y. , & Todorov, A. (2013). The neural dynamics of updating person impressions. Social Cognitive and Affective Neuroscience, 8(6), 623–631. 10.1093/scan/nss040 22490923 PMC3739907

[bjso70123-bib-0035] Mende‐Siedlecki, P. , & Todorov, A. (2016). Neural dissociations between meaningful and mere inconsistency in impression updating. Social Cognitive and Affective Neuroscience, 11(9), 1489–1500. 10.1093/scan/nsw058 27217118 PMC5015807

[bjso70123-bib-0036] Menne‐Lothmann, C. , Viechtbauer, W. , Höhn, P. , Kasanova, Z. , Haller, S. P. , Drukker, M. , Van Os, J. , Wichers, M. , & Lau, J. Y. F. (2014). How to boost positive interpretations? A meta‐analysis of the effectiveness of cognitive bias modification for interpretation. PLoS One, 9(6), e100925. 10.1371/journal.pone.0100925 24968234 PMC4072710

[bjso70123-bib-0037] Mercier, H. , & Sperber, D. (2011). Why do humans reason? Arguments for an argumentative theory. Behavioral and Brain Sciences, 34(2), 57–74. 10.1017/S0140525X10000968 21447233

[bjso70123-bib-0038] Mestdagh, M. , Verdonck, S. , Piot, M. , Niemeijer, K. , Kilani, G. , Tuerlinckx, F. , Kuppens, P. , & Dejonckheere, E. (2023). M‐path: An easy‐to‐use and highly tailorable platform for ecological momentary assessment and intervention in behavioral research and clinical practice. Frontiers in Digital Health, 5, 1182175. 10.3389/fdgth.2023.1182175 37920867 PMC10619650

[bjso70123-bib-0039] Moskowitz, G. B. , Okten, I. O. , & Schneid, E. (2022). The updating of first impressions. In The handbook of impression formation. Routledge.

[bjso70123-bib-0040] Nelson, J. D. (2008). Towards a rational theory of human information acquisition. In N. Chater & M. Oaksford (Eds.), The probabilistic mind (1st ed., pp. 143–164). Oxford University Press. 10.1093/acprof:oso/9780199216093.003.0007

[bjso70123-bib-0041] Nickerson, R. S. (1998). Confirmation bias: A ubiquitous phenomenon in many guises. Review of General Psychology, 2(2), 175–220. 10.1037/1089-2680.2.2.175

[bjso70123-bib-0042] Peters, U. (2022). What is the function of confirmation bias? Erkenntnis, 87(3), 1351–1376. 10.1007/s10670-020-00252-1

[bjso70123-bib-0043] Rabin, M. , & Schrag, J. L. (1999). First impressions matter: A model of confirmatory bias*. The Quarterly Journal of Economics, 114(1), 37–82. 10.1162/003355399555945

[bjso70123-bib-0044] Reeder, G. D. , & Brewer, M. B. (1979). A schematic model of dispositional attribution in interpersonal perception. Psychological Review, 86(1), 61–79. 10.1037/0033-295X.86.1.61

[bjso70123-bib-0045] Richey, M. H. , Mcclelland, L. , & Shimkunas, A. M. (1967). Relative influence of positive and negative information in impression formation and persistence. Journal of Personality and Social Psychology, 6(3), 322–327. 6075214 10.1037/h0024734

[bjso70123-bib-0046] Rigoux, L. , Stephan, K. E. , Friston, K. J. , & Daunizeau, J. (2014). Bayesian model selection for group studies—Revisited. NeuroImage, 84, 971–985. 10.1016/j.neuroimage.2013.08.065 24018303

[bjso70123-bib-0047] Rothbart, M. , & Park, B. (1986). On the confirmability and disconfirmability of trait concepts. Journal of Personality and Social Psychology, 50(1), 131–142. 10.1037/0022-3514.50.1.131 17352608

[bjso70123-bib-0048] Rozin, P. , & Royzman, E. B. (2001). Negativity bias, negativity dominance, and contagion. Personality and Social Psychology Review, 5(4), 296–320. 10.1207/S15327957PSPR0504_2

[bjso70123-bib-0049] Schwarz, N. , & Bless, H. (1992). Constructing reality and its alternatives: An inclusion/exclusion model of assimilation and contrast effects in social judgment. In The construction of social judgments (pp. 217–245). Lawrence Erlbaum Associates, Inc.

[bjso70123-bib-0050] Sinclair, S. , Lowery, B. S. , Hardin, C. D. , & Colangelo, A. (2005). Social tuning of automatic racial attitudes: The role of affiliative motivation. Journal of Personality and Social Psychology, 89(4), 583–592. 10.1037/0022-3514.89.4.583 16287420

[bjso70123-bib-0051] Skowronski, J. , & Carlston, D. (1987). Social judgment and social memory: The role of Cue Diagnosticity in negativity, positivity, and extremity biases. Journal of Personality and Social Psychology, 52, 689–699. 10.1037/0022-3514.52.4.689

[bjso70123-bib-0052] Skowronski, J. J. , & Carlston, D. E. (1989). Negativity and extremity biases in impression formation: A review of explanations. Psychological Bulletin, 105(1), 131–142. 10.1037/0033-2909.105.1.131

[bjso70123-bib-0053] Smith, E. R. , & Semin, G. R. (2007). Situated social cognition. Current Directions in Psychological Science, 16(3), 132–135. 10.1111/j.1467-8721.2007.00490.x

[bjso70123-bib-0054] Stephan, K. E. , Penny, W. D. , Daunizeau, J. , Moran, R. J. , & Friston, K. J. (2009). Bayesian model selection for group studies. NeuroImage, 46(4), 1004–1017. 10.1016/j.neuroimage.2009.03.025 19306932 PMC2703732

[bjso70123-bib-0055] Taber, C. S. , & Lodge, M. (2006). Motivated skepticism in the evaluation of political beliefs. American Journal of Political Science, 50(3), 755–769. 10.1111/j.1540-5907.2006.00214.x

[bjso70123-bib-0056] Torres, S. C. , Gracia Laso, D. I. , Minissi, M. E. , Maddalon, L. , Chicchi Giglioli, I. A. , & Alcañiz, M. (2024). Social signal processing in affective virtual reality: Human‐shaped agents increase Electrodermal activity in an elicited negative environment. Cyberpsychology, Behavior and Social Networking, 27(4), 268–274. 10.1089/cyber.2023.0273 38394167

[bjso70123-bib-0057] Trafimow, D. , & Schneider, D. J. (1994). The effects of behavioral, situational, and person information on different attribution judgments. Journal of Experimental Social Psychology, 30(4), 351–369. 10.1006/jesp.1994.1017

[bjso70123-bib-0058] Trope, Y. , & Bassok, M. (1982). Confirmatory and diagnosing strategies in social information gathering. Journal of Personality and Social Psychology, 43(1), 22–34. 10.1037/0022-3514.43.1.22

[bjso70123-bib-0059] Trouche, E. , Johansson, P. , Hall, L. , & Mercier, H. (2016). The selective laziness of reasoning. Cognitive Science, 40(8), 2122–2136. 10.1111/cogs.12303 26452437

[bjso70123-bib-0060] Tversky, A. , & Kahneman, D. (1974). Judgment under uncertainty: Heuristics and biases. Science, 185(4157), 1124–1131. 17835457 10.1126/science.185.4157.1124

[bjso70123-bib-0061] Uleman, J. , & Kressel, L. M. (2013). A brief history of theory and research on impression formation. In The Oxford handbook of social cognition (pp. 53–73). Oxford University Press.

[bjso70123-bib-0062] Van Overwalle, F. , & Labiouse, C. (2004). A recurrent connectionist model of person impression formation. Personality and Social Psychology Review, 8(1), 28–61. 10.1207/S15327957PSPR0801_2 15121539

[bjso70123-bib-0063] Vedejová, D. , & Čavojová, V. (2022). Confirmation bias in information search, interpretation, and memory recall: Evidence from reasoning about four controversial topics. Thinking & Reasoning, 28(1), 1–28. 10.1080/13546783.2021.1891967

[bjso70123-bib-0064] Voichek, G. , & Novemsky, N. (2024). Positive contrast scope insensitivity. Journal of Consumer Research, 25, ucae052. 10.1093/jcr/ucae052

[bjso70123-bib-0065] Williams, M. J. , & Tiedens, L. Z. (2016). The subtle suspension of backlash: A meta‐analysis of penalties for women's implicit and explicit dominance behavior. Psychological Bulletin, 142(2), 165–197. 10.1037/bul0000039 26689089

[bjso70123-bib-0066] Williamson, J. B. , Drago, V. , Harciarek, M. , Falchook, A. D. , Wargovich, B. A. , & Heilman, K. M. (2019). Chronological effects of emotional valence on the self‐selected retrieval of autobiographical memories. Cognitive and Behavioral Neurology, 32(1), 11–15. 10.1097/WNN.0000000000000183 30896572

[bjso70123-bib-0067] Wojciszke, B. , Brycz, H. , & Borkenau, P. (1993). Effects of information content and evaluative extremity on positivity and negativity biases. Journal of Personality and Social Psychology, 64(3), 327–335. 10.1037/0022-3514.64.3.327

[bjso70123-bib-0068] Wood, C. , & Hutter, R. R. C. (2011). The importance of being emergent: A theoretical exploration of impression formation in novel social category conjunctions. Social and Personality Psychology Compass, 5(6), 321–332. 10.1111/j.1751-9004.2011.00353.x

[bjso70123-bib-0069] Xie, S. Y. , Thai, S. , & Hehman, E. (2023). Everyday perceiver‐context influences on impression formation: No evidence of consistent effects. Personality and Social Psychology Bulletin, 49(6), 955–968. 10.1177/01461672221085088 35471126 PMC10225998

[bjso70123-bib-0070] Ybarra, O. , Chan, E. , & Park, D. (2001). Young and old adults' concerns about morality and competence. Motivation and Emotion, 25(2), 85–100. 10.1023/A:1010633908298

[bjso70123-bib-0071] Yeates, P. , Moreau, M. , & Eva, K. (2015). Are examiners' judgments in OSCE‐style assessments influenced by contrast effects? Academic Medicine: Journal of the Association of American Medical Colleges, 90(7), 975–980. 10.1097/ACM.0000000000000650 25629945

[bjso70123-bib-0072] Ziegler, J. , McCaughey, L. , & Fiedler, K. (2026). Flexible Diagnosticity in person impression formation: An integrative framework. Personality and Social Psychology Bulletin, 15, 5990. 10.1177/01461672251405990 41506721

