# Supplementary material for: Contrast bias, not confirmation bias, shapes daily impression updating across social and non‐social targets
Source: Br J Soc Psychol. 2026 Aug 7;65(4):e70123. doi: 10.1111/bjso.70123 (PMC13451568; doi:10.1111/bjso.70123)
Supplement: Supplementary file 1 [file BJSO-65-0-s001.zip › bjso70123-sup-0005-SupinfoS4.docx]

**Supplemental material 4**

*The following two figures are the model selection results from both studies with Recency Effect Model added.*

*Model 1: Baseline Bayesian Model.*

*Model 2: Bayesian Bias Model.*

*Model 3: Bayesian Triggered Model*

*Model 4: Cumulative Bias Model (6 days memory, no prior)*

*Model 5: Cumulative Bias Model (6 days memory, no prior, update only when there is ratio change)*

*Model 6: Cumulative Bias Model (5 days memory, no prior)*

*Model 7: Cumulative Bias Model (4 days memory, no prior)*

*Model 8: Cumulative Bias Model (3days memory, no prior)*

*Model 9: Cumulative Bias Model (2 days memory, no prior)*

*Model 10: Cumulative Bias Model (1 days memory, no prior)*

*Model 11: Recency effect Model*

Study 1:

Study 2:
