# Supplementary material for: Contrast bias, not confirmation bias, shapes daily impression updating across social and non‐social targets
Source: Br J Soc Psychol. 2026 Aug 7;65(4):e70123. doi: 10.1111/bjso.70123 (PMC13451568; doi:10.1111/bjso.70123)
Supplement: Supplementary file 1 [file BJSO-65-0-s001.zip › bjso70123-sup-0002-SupinfoS1.docx]

**Supplemental Material 1**

**Stimulus Pretesting**

We pretested 42 behaviour combinations with 105 participants via Prolific. Each combination consisted of three behaviour statements categorized a priori as negative (B), positive (G), or neutral (N). Participants rated each combination on a 0–100 valence scale (0 = very negative, 100 = very positive). After removing missing responses, 2,205 ratings were retained for analysis.

Median ratings confirmed separation between negative and positive categories: all-negative combinations (BBB) received the lowest ratings (*Mdn* = 6, *IQR* [1–14], *M* = 11.6, *SD* = 15.3), while all-positive combinations (GGG) received high ratings (*Mdn* = 81, *IQR* [72–91], *M* = 77.6, *SD* = 18.0). Mixed combinations fell between these extremes, with ratings monotonically decreasing as the number of negative statements increased.

We removed the neutral category from the main studies based on pretest results. All-neutral combinations (NNN) received ratings (*Mdn* = 81, *IQR* [70–91], *M* = 78.4, *SD* = 16.7) that were essentially indistinguishable from all-positive combinations (GGG; *Mdn* = 81, *M* = 77.6), rather than falling near the midpoint of the scale. This indicated that participants interpreted neutral behaviours as mildly positive by default, and that the operational definition of "neutral" did not yield a distinct third category. We therefore focused the main studies on the positive–negative contrast.

Within-category variability was substantial (*SDs* 15–18), but between-category separation was consistent across the distribution, as reflected in the non-overlapping interquartile ranges for BBB versus GGG/NNN.
