# Supplementary material for: Contrast bias, not confirmation bias, shapes daily impression updating across social and non‐social targets
Source: Br J Soc Psychol. 2026 Aug 7;65(4):e70123. doi: 10.1111/bjso.70123 (PMC13451568; doi:10.1111/bjso.70123)
Supplement: Supplementary file 1 [file BJSO-65-0-s001.zip › bjso70123-sup-0003-SupinfoS2.docx]

**Supplemental Material 2**

For mathematical convenience, we model people’s prior impression as a beta distribution on x, as follows:

$f\left( x;a,b \right)=beta\left( a,b \right)=\frac{x^{a-1}\left( 1-x \right)^{b-1}}{B\left( a,b \right)}$, where $B\left( a,b \right)= \int x^{a-1} \left( 1-x \right)^{b-1} dx$	(1)

The prior impression has two parameters, *a* and *b*, where *a* is the number of positive pieces of information one has observed so far, and b represents the number of negative behaviors one has observed so far. The mean of the beta distribution, calculated as $E\left[ x \right]=a/{a+b}$ (the expectation of the hidden trait x), represents the proportion of positive pieces of information among all pieces of information. This can be interpreted as the perceived "goodness" of the target.

People do not have direct access to the hidden trait x. Rather, they receive positive or negative pieces of information that depend upon the hidden trait. More precisely, we assume that people are more likely to receive positive information if x increases. This can be modeled by stating that the number of positive pieces of information (among all types of information) follows a binomial distribution, with probability x. Let 𝑛 represent the total number of pieces of information (either positive or negative), and let 𝑘 represent the number of positive pieces of information among all the pieces of information. The likelihood of receiving k positive pieces of information out of n total pieces is written as:

$P\left( k \right|n, x)=\binom{n}{k} x^{k}\left( 1-x \right)^{n-k}$	(2)

This simply states that the hidden trait x controls the rate of positive information that people receive when forming an impression about the target.

The impression update rule uses Bayes’ Theorem to combine the prior distribution with the likelihood of the observed data – k positive pieces of information out of n in total – to calculate the posterior distribution of $x$:

$P\left( x | k,n \right)=\frac{P\left( k | x, n \right)P(x)}{P(k, n)}$	(3)

Here, $P\left( x | k,n \right)$ is the posterior distribution, which presents the updated belief about x after observing the data. $P(k, n)$ is the so-called marginal likelihood, which normalizes the posterior distribution. Replacing Equations 1 and 2 into Equation 3 yields:

$P\left( x | k,n \right)=\frac{[\binom{n}{k} x^{k}\left( 1-x \right)^{n-k}][\frac{x^{a-1}\left( 1-x \right)^{b-1}}{B\left( a,b \right)}]}{P(k,n)}$	(4)

where	$P\left( k,n \right)=\int_{0}^{1} P\left( k | x \right)P\left( x \right)dx$

$=\int_{0}^{1} \binom{n}{k} x^{k}\left( 1-x \right)^{n-k}\frac{x^{a-1}\left( 1-x \right)^{b-1}}{B\left( a,b \right)}$dx

$=\binom{n}{k}*\frac{1}{B\left( a,b \right)}\int_{0}^{1} x^{k+a-1}\left( 1-x \right)^{n-k+b-1}dx$

$$=\binom{n}{k}*\frac{B(k+a, n-k+b)}{B(a,b)}$$

In turn, the posterior distribution can be written as:

$P\left( x | k,n \right)=\frac{\binom{n}{k}x^{k+a-1}\left( 1-x \right)^{n-k+b-1}}{B\left( a,b \right)\binom{n}{k}\frac{B(k+a, n-k+b)}{B(a,b)}}=\frac{x^{k+a-1}\left( 1-x \right)^{n-k+b-1}}{B(k+a, n-k+b)}$	(5)

This implies that the posterior distribution also has a Beta functional form:

$P\left( x | k,n \right)=Beta(k+a, n-k+b)$	(6)

As illustrated above, the reason why the posterior distribution remains a Beta distribution is that the binomial distribution is the conjugate of the Beta distribution. This conjugacy implies that when applying Bayes’ rule to incorporate new information (modeled as a binomial distribution), the resulting posterior distribution continues to follow a Beta distribution. If the process goes on, the posterior distribution becomes the new prior for subsequent observations, which results in a simple analytical iterative impression update rule. Let $G_{i}$ and $B_{i}$ be the number of good and bad pieces of information at time $i$, and $a_{i}$, and $b_{i}$ their associated cumulated sum up to time $i$. At time $i+1$, the posterior distribution can be written as:

$P\left( x | G_{1}\ldots G_{i+1},B_{1}\ldots B_{i+1} \right)= Beta(a_{i+1},b_{i+1})$,

$=Beta(a_{i}+G_{+1}, b_{i}+B_{i+1})$	(7)
