# Supplementary material for: Contrast bias, not confirmation bias, shapes daily impression updating across social and non‐social targets
Source: Br J Soc Psychol. 2026 Aug 7;65(4):e70123. doi: 10.1111/bjso.70123 (PMC13451568; doi:10.1111/bjso.70123)
Supplement: Supplementary file 1 [file BJSO-65-0-s001.zip › bjso70123-sup-0004-SupinfoS3.docx]

**Supplemental material 3**

We examined whether decisions provided information beyond impression ratings. For each study, we fit a logistic mixed-effects model predicting decisions (positive vs. negative) from impression rating, target type (social vs. non-social), and their interaction, with a random intercept by participant.

In both studies, impression rating strongly predicted decisions (Study 1: β = 0.21, SE = 0.02, z = 8.95, p < .001; Study 2: β = 0.17, SE = 0.02, z = 11.04, p < .001). Target type had a main effect on decisions (Study 1: β = 2.84, p = .024; Study 2: β = 2.59, p = .007), with social targets receiving more positive decisions than non-social targets at equivalent impression levels — likely reflecting different baseline decision thresholds across the two contexts (hiring vs. booking). However, the impression × target interaction was not significant in either study (Study 1: β = -0.05, p = .062; Study 2: β = -0.03, p = .138), indicating that the impression-decision relationship was comparable across target types.

Together, these analyses indicate that decisions tracked impression ratings closely and did not provide systematic information beyond what impressions already capture. We therefore focus on impression ratings as the primary outcome measure in the main text.
